# The New National Quarantine Law

**Published:** 1888-09

**Authors:** 


### EDITORIAL.

### THE NEW NATIONAL QUARANTINE LAW.

The outbreak of yellow fever at several of our Gulf and South Atlantic ports has stimulated Congress to another spasmodic effort looking to the protection of the public from contagious and infectious diseases. The fearful epidemic in the Mississippi Valley ten years ago was followed by legislation creating a National Board of Health, and conferring upon it powers, and providing it with means to combat the dangers which then menaced the whole country. The Board formulated rules and planned measures which, in their operation, have secured to the country almost complete immunity up to the present season. It is true that when the emergency had passed and the threatened dangers diminished, the support to the National Board was withdrawn, and the machinery which it had created and the methods which it had devised were adopted and used with but little change by the Marine Hospital Service. The National Board still exists and its duties and powers are adequate to meet the emergencies. It is, however, not a political body, and its machinery can not be used for the purposes of free trade, nor for protection, only so far as human life and health are concerned. There is, therefore, for its support, no strong motive which appeals to the average Congressman. It is a gratification to know that something has been done, and that in the right direction. Provision is made in the act which has recently received the approval of the President, and which will be found elsewhere in our columns, for the establishment of quarantine stations at various points along the Atlantic, Gulf, and Pacific coasts to which infected ships may be sent for disinfection; in other words, "ship hospitals." This was one of the most important measures inaugurated by the National Board of Health in 1879. It is almost certain that in time the whole subject of maritime quarantine will be placed under the direction of the General Government as it ought to be. The interior States are quite as much interested in this matter as those along the seaboard; in some instances, indeed, the interest of the States remote from the sea is greater than that of those at which vessels discharge their cargoes of freight and immigrants. This is apparent to any one at all familiar with the history of the epidemics which have invaded this country from abroad. The temporary control of this matter by the Marine Hospital Service may be a convenience, but there should be devised some more special and appropriate bureau or department, with well-defined powers and duties, and provided with the means necessary to not only cope with infection from abroad, but also to protect individual States from dangers that may threaten them from neighboring States. Millions are appropriated to the scientific and economic interests of our people, and we hail with pleasure this wise fact, but it would be a mark of still greater wisdom to regard human health and human life as a large part of the national wealth, and to give for their protection something of the same thought and means that is done for the preservation of our cattle, the development of our varied industries, and the scientific investigation of our natural history.

This will only be reached when there shall be a department of public health, or at least a bureau which shall be charged with this special subject. So far, the questions of vital statistics, including the life-tendencies, the morbidity, mortality, etc., have been under the direction of the Department of the Interior, and appear once in ten years with the innumerable other facts of the census reports, the accuracy of many of which is open to question. Protection from epidemics has hardly occupied the attention of our National Legislature excepting in the presence of the scourge. Such action has always been regarded as provisional. It is time that we should give to this subject the same attention that we do to national dangers of other kinds. "In peace prepare for war."

